# Structural Basis for Feed-Forward Transcriptional Regulation of Membrane Lipid Homeostasis in *Staphylococcus aureus*

**DOI:** 10.1371/journal.ppat.1003108

**Published:** 2013-01-03

**Authors:** Daniela Albanesi, Georgina Reh, Marcelo E. Guerin, Francis Schaeffer, Michel Debarbouille, Alejandro Buschiazzo, Gustavo E. Schujman, Diego de Mendoza, Pedro M. Alzari

**Affiliations:** 1 Institut Pasteur, Unité de Microbiologie Structurale (CNRS UMR 3528), Paris, France; 2 Instituto de Biología Molecular y Celular de Rosario (IBR), Facultad de Ciencias Bioquímicas y Farmacéuticas, Universidad Nacional de Rosario, Rosario, Argentina; 3 Unidad de Biofisica, Centro Mixto CSIC-UPV/EHU, Leioa, Bizkaia, Spain; 4 Departamento de Bioquímica, Universidad del País Vasco, Bilbao, Spain; 5 IKERBASQUE, Basque Foundation for Science, Bilbao, Spain; 6 Institut Pasteur, Unité de Biologie des Bactéries Pathogènes à Gram Positif (CNRS ERL 3526), Paris, France; University of Michigan, United States of America

## Abstract

The biosynthesis of membrane lipids is an essential pathway for virtually all bacteria. Despite its potential importance for the development of novel antibiotics, little is known about the underlying signaling mechanisms that allow bacteria to control their membrane lipid composition within narrow limits. Recent studies disclosed an elaborate feed-forward system that senses the levels of malonyl-CoA and modulates the transcription of genes that mediate fatty acid and phospholipid synthesis in many Gram-positive bacteria including several human pathogens. A key component of this network is FapR, a transcriptional regulator that binds malonyl-CoA, but whose mode of action remains enigmatic. We report here the crystal structures of FapR from *Staphylococcus aureus* (*Sa*FapR) in three relevant states of its regulation cycle. The repressor-DNA complex reveals that the operator binds two *Sa*FapR homodimers with different affinities, involving sequence-specific contacts from the helix-turn-helix motifs to the major and minor grooves of DNA. In contrast with the elongated conformation observed for the DNA-bound FapR homodimer, binding of malonyl-CoA stabilizes a different, more compact, quaternary arrangement of the repressor, in which the two DNA-binding domains are attached to either side of the central thioesterase-like domain, resulting in a non-productive overall conformation that precludes DNA binding. The structural transition between the DNA-bound and malonyl-CoA-bound states of *Sa*FapR involves substantial changes and large (>30 Å) inter-domain movements; however, both conformational states can be populated by the ligand-free repressor species, as confirmed by the structure of *Sa*FapR in two distinct crystal forms. Disruption of the ability of *Sa*FapR to monitor malonyl-CoA compromises cell growth, revealing the essentiality of membrane lipid homeostasis for *S. aureus* survival and uncovering novel opportunities for the development of antibiotics against this major human pathogen.

## Author Summary

An opportunistic Gram-positive pathogen, *Staphylococcus aureus* is a major threat to humans and animals, being responsible for a variety of infections ranging from mild superficial to severe infections such as infective endocarditis, septic arthritis, osteomyelitis and sepsis. The increasing resistance of *S. aureus* against most current antibiotics emphasizes the need to develop new approaches to control this important pathogen. The lipid biosynthetic pathway is one appealing target actively pursued to develop anti-Staphylococcal agents. Despite its potential biomedical importance, however, little is known about the signaling mechanisms that allow *S. aureus* to control its phospholipid content. In order to shed light on this fundamental mechanism, we studied *S. aureus* FapR (*Sa*FapR) a transcription factor that senses the levels of malonyl-CoA, a key intermediate in fatty acid biosynthesis, and modulates the expression of genes involved in fatty acid and phospholipid biosynthesis. Our studies of *Sa*FapR uncovered the mechanistic basis of a complex biological switch that controls membrane lipid homeostasis in *S. aureus*. We also discovered that disruption of the ability of *Sa*FapR to recognize malonyl-CoA, the ligand that controls *Sa*FapR binding to DNA, compromises *S. aureus* viability, thus revealing new opportunities for the development of antibiotics against this major human pathogen.

## Introduction

The cell membrane is an essential structure to bacteria. It primarily consists of a fluid phospholipid bilayer in which a variety of proteins are embedded. Most steps involved in phospholipid biosynthesis are therefore explored as targets for designing new antibacterial drugs [1]. The central events in the building of phospholipids enclose the biosynthesis of fatty acids, which are the most energetically expensive lipid components, by the type II fatty acid synthase (FASII) on the cytoplasmic side of the membrane, and subsequent delivery of the latter to the membrane-bound glycerol-phosphate acyltransferases (Fig. S1). Due to the vital role of the membrane lipid bilayer, bacteria have evolved sophisticated mechanisms to finely control the expression of the genes responsible for the metabolism of phospholipids [2].

Transcriptional regulation of bacterial lipid biosynthetic genes is poorly understood at the molecular level. Indeed, the only two well-documented examples are probably those of the transcription factors FadR and DesT, which regulate the biosynthesis of unsaturated fatty acids (UFA) in *Escherichia coli* and *Pseudomonas aeruginosa*, respectively. FadR was discovered as a repressor of the β-oxidation regulon [3], [4] and subsequently found to also activate the transcription of *fabA* and *fabB*, two essential genes for the biosynthesis of UFA [5]–[7]. Binding of FadR to its DNA operator is antagonized by different long-chain acyl- CoAs [8]–[11], in agreement with the proposed structural model [12]–[14]. DesT is a TetR-like repressor that primarily controls the expression of the *desCB* operon, which encodes the components of an oxidative fatty acid desaturase, and secondarily controls the expression of the *fabAB* operon of *Pseudomonas aeruginosa* [15]. DesT binds either saturated or unsaturated acyl-CoAs, which respectively prevent or enhance its DNA-binding properties [16], thus allowing the repressor to differentially respond to alternate ligand shapes [17].

Among the known regulatory mechanisms that control lipid synthesis in bacteria, the *Bacillus subtilis* Fap system is unique in that the regulated final products, fatty acids and phospholipids, are controlled by a metabolite required at the beginning of the fatty acid biosynthetic pathway, malonyl-CoA [18], [19]. To monitor the levels of this metabolite, bacteria employ the FapR protein [20], which is highly conserved among several Gram-positive pathogens. FapR has been shown to globally repress the expression of the genes from the *fap* regulon (Fig. S1) encoding the soluble FASII system as well as two key enzymes that interface this pathway with the synthesis of phospholipid molecules [18].

Like most transcriptional regulators in bacteria, FapR is a homodimeric repressor [20]. Each protomer consists of a N-terminal DNA-binding domain (DBD) harboring a classical helix-turn-helix motif connected through a linker α-helix (α_L_) to a C-terminal effector-binding domain (EBD). We have previously determined the structure of a truncated form of FapR from *Bacillus subtilis* (*Bs*FapR), which included the linker α-helix and the EBD but lacked the DBD [20]. The EBD folds into a symmetric dimer displaying a ‘hot-dog’ architecture, with two central α-helices surrounded by an extended twelve-stranded β-sheet. A similar fold has been found in many homodimeric acyl-CoA-binding enzymes [21], [22] involved in fatty acid biosynthesis and metabolism [23], [24]. However, the bacterial transcriptional regulator FapR appears to be so far the only well-characterized protein family to have recruited the ‘hot-dog’ fold for a non-enzymatic function.

The structure of truncated FapR bound to malonyl-CoA revealed structural changes in some ligand-binding loops of the EBD, and it was suggested that these changes could propagate to the missing DNA-binding domains to impair their productive association for DNA binding [20]. However, the actual mechanisms involved remain largely unknown in the absence of detailed structural information of the full-length repressor and its complex with DNA. Here, we report the structural characterization of full-length FapR from *Staphylococcus aureus* (*Sa*FapR), a major Gram-positive pathogen causing severe human infections [25]–[27]. The crystal structures of *Sa*FapR have been obtained for the protein alone and for the complexes with the cognate DNA operator and the effector molecule, malonyl-CoA, providing important mechanistic insights into the mode of action of this transcriptional regulator. We further demonstrate that structure-based *Sa*FapR mutants interfering with malonyl-CoA binding are lethal for *S. aureus*. These data show that membrane lipid homeostasis is essential for *S. aureus* survival and highlights this regulatory mechanism as an attractive target to develop new antibiotics.

## Results

### FapR senses malonyl-CoA and regulates lipid biosynthesis in *S. aureus*

The *S. aureus fap* regulon is organized as in *B. subtilis* [18], except for two missing genes (*yhfC* and *fabHB*) (Table S1). Electrophoretic mobility shift assays revealed that *Sa*FapR binds to its own promoter (P*fapR*) and that malonyl-CoA specifically disrupts the repressor-operator complex (Fig. S2). Furthermore, the unlinked genes *plsX* and *fabH* from the *fap* regulon are upregulated in two distinct *S. aureus* strains lacking the repressor (Fig. S3A) and *Sa*FapR is able to complement a *B. subtilis fapR* mutant strain (Fig. S3B). These results clearly demonstrate that *Sa*FapR conserves the same regulatory function originally described for the *B. subtilis* orthologue [20].

### The *Sa*FapR-DNA complex

**Figure 1:**
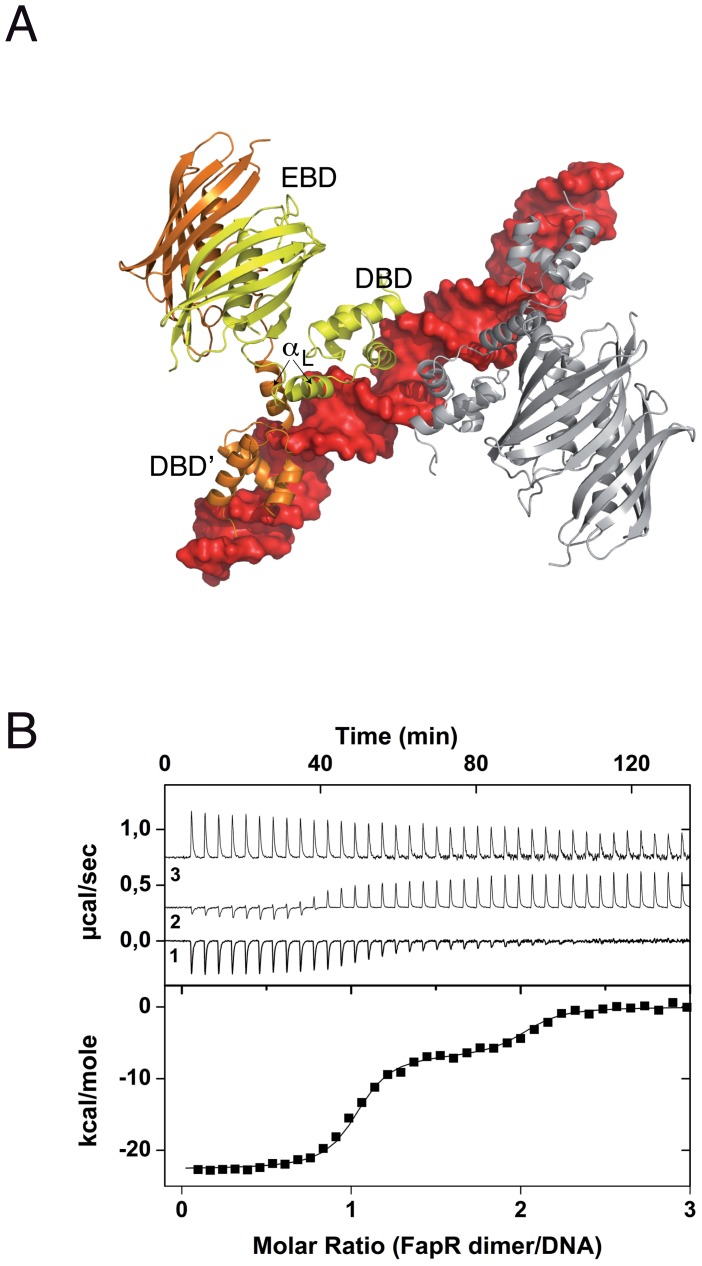
Overall structure of the *Sa*FapR-operator complex. (**A**) Surface representation of the DNA operator (in red) with two bound FapR homodimers looking down the non-crystallographic two-fold symmetry axis. For one homodimeric repressor (in yellow and orange) the DNA-binding domains (DBDs), the linker helix α_L_ and the dimeric effector-binding domain (DBD) are indicated. (**B**) ITC study of *Sa*FapR binding to the P*fapR* operator at 25°C. The top panel shows the raw heat signal for 6 µl injections of a 68 µM solution of *Sa*FapR dimer into a 4 µM solution of the 40 bp DNA oligonucleotide (curve 1 obtained by subtraction of the *Sa*FapR dilution energy curve 3 from the raw titration curve 2). The bottom panel shows the integrated injection heats after normalization fitted with a sequential binding model. Two *Sa*FapR dimers bind the operator, with parameters (*K_d_*_,I_ = 0.5±0.1 nM, *ΔH*°_I_ = −22.5±0.2 kcal/mol, *TΔS*°_I_ = −9.8±0.2, kcal/mol) and (*K_d_*_,II_ = 51±8 nM, *ΔH*°_II_ = −6.95±0.2 kcal/mol, *TΔS*°_II_ = 3.0±0.3 kcal/mol).

The crystal structure of *Sa*FapR in complex with a 40-bp oligonucleotide comprising the P*fapR* promoter was determined at 3.15 Å resolution (Table 1). Two *Sa*FapR homodimers were observed to bind to a single DNA molecule in the crystal (Fig. 1A). This observation is in agreement with isothermal titration calorimetry (ITC) studies of protein-DNA interactions, which showed a complex isotherm with a large exothermic component and two distinct binding reactions (Fig. 1B). Sequence analysis of the promoters of the *fap* regulon from *B. subtilis* [18] and *S. aureus* (Fig. S4) revealed the presence of a conserved inverted repeat. Our previous DNAse I footprinting analyses performed with *Bs*FapR on both strands of the *B. subtilis fapR* promoter demonstrated that this symmetric element covers half of the DNAse protected region [20]. Interestingly, this region corresponds to the recognition site of one *Sa*FapR homodimer in the crystal, suggesting a sequential mechanism of binding. Indeed, a sequential binding model fits well the observed isotherm (Fig. 1B), indicating that the two *Sa*FapR homodimers bind the operator with nanomolar dissociation constants of 0.5±0.1 nM for the first and 51±8 nM for the second binding reactions. In the crystal structure of the complex, however, the two *Sa*FapR homodimers are related by a local two-fold symmetry axis, and the DNA molecule is bound in a 50∶50 orientation, as confirmed by determining the crystal structure of *Sa*FapR in complex with an asymmetrically Br-labelled oligonucleotide using the Br anomalous scattering signal (data not shown).

**Table 1:** Data collection, phasing and refinement statistics.

	*Sa*FapR-DNA	*Sa*FapR-malonyl-CoA	*Sa*FapR (form 1)	*Sa*FapR (form 2)
**Data collection**				
Space group	C222_1_	P2_1_	H32	P3_1_21
Cell dimensions				
*a*, *b*, *c* (Å)	114.69, 249.94, 179.21	53.38, 68.5, 61.16	116.58, 116.58, 123.23	110.52, 110.52, 115.11
α, β, γ (°)	90, 90, 90	90, 111.3, 90	90, 90, 120	90, 90, 120
Resolution (Å)	35-3.15 (3.32-3.15)	44-1.9 (2-1.9)	47-2.4 (2.53-2.4)	48-2.6 (2.74-2.6)
*R*_sym_ or *R*_merge_	0.099 (0.572)	0.043 (0.349)	0.060 (0.634)	0.061 (0.426)
*I*/σ*I*	24.3 (2.4)	16.4 (3.6)	15.6 (2.5)	16.7 (3.4)
Completeness (%)	98.2 (93.7)	99.3 (100)	99.9 (100)	99.7 (98.5)
Redundancy	16.0 (4.9)	3.7 (3.7)	5.5 (5.6)	3.7 (3.6)
**Refinement**				
Resolution (Å)	3.15	1.9	2.4	2.6
No. reflections	41820	30557	12145	24146
*R*_work_/*R*_free_	0.186/0.201	0.198/0.235	0.196/0.246	0.185/0.211
No. atoms				
Protein	6040	2538	1460	2431
Ligand/ion	1634	98	25	9
Water	-	150	20	52
*B*-factors				
Protein	104	42	63	47
Ligand/Ion	149	41	67	57
Water	-	28	34	18
Ramachandran outliers^1^ (%)	0.5	0	0	0
Ramachandran favored^1^ (%)	95.8	98.1	97.8	97.0
R.m.s. deviations				
Bond lengths (Å)	0.007	0.012	0.012	0.017
Bond angles (°)	1.14	1.39	1.64	1.61

Values in parentheses are for highest-resolution shell.

1 According to the MolProbity server [53].

Each protein homodimer exhibits an elongated asymmetric conformation in the crystal structure, in which the two DNA-bound DBDs are structurally detached from the central dimeric ‘hot-dog’ EBD. Indeed, the positioning of the EBD (stabilized by crystal packing contacts) relative to the DBD-DNA complex requires partial unwinding of the C-terminal end of α_L_, as confirmed by comparing the structures of the ligand-free and DNA-bound forms of *Sa*FapR (see below). The amphipatic linker helix α_L_ plays a major role in stabilizing the molecular architecture of the *Sa*FapR-DNA complex. Helix α_L_ interacts with α_L_′ from the second protomer mainly through its exposed hydrophobic face (including residues Ile59, Val62 and Ala63; Fig. S5A). This dimerization region is further stabilized by hydrophobic contacts (Phe26) and hydrophilic interactions between α_L_ and the α1–α2 connecting loop from both DBDs. In addition, the guanidinium group of Arg59 gets engaged in electrostatic interactions with the main-chain carbonyl groups of Pro25 and Ile27, and the hydroxyl group of Tyr67 is within hydrogen bonding distance to the main-chain of Ser23 at the end of α1′ on the second protomer (Fig. S5A). Altogether this contact network largely contributes to the correct relative positioning of the two helix-turn-helix motifs on the DNA major groove.

**Figure 2:**
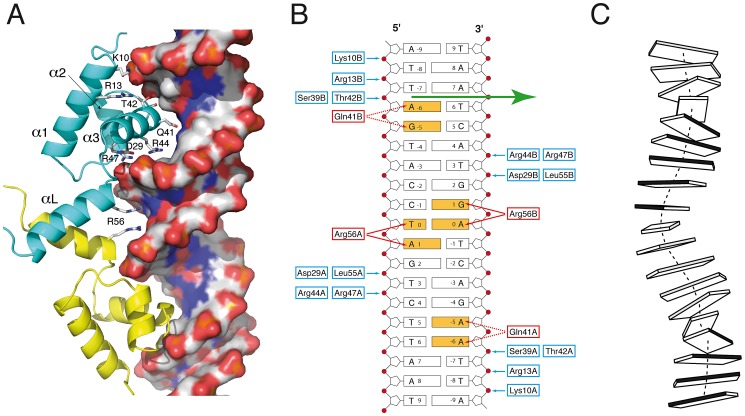
*Sa*FapR-DNA interactions. (**A**) Promoter recognition by the *Sa*FapR DNA-binding domain. Protein residues making hydrogen-bonding interactions with specific bases (Gln41, Arg56) or with the phosphate backbone are indicated. The DNA double-helix is depicted in solvent accessible surface representation and colored according to the mapped electrostatic potential (negative charge in red, positive in blue). (**B**) Schematic representation of protein-DNA hydrogen-bonding interactions for one FapR homodimer. Protein residues involved in base-specific hydrogen-bonding interactions are colored red and those involved in phosphate hydrogen-bonding interactions are blue; the specifically recognized bases are orange. In the crystal structure, the DNA duplex exists 50∶50 in the two possible orientations, and the figure shows the 5′ to 3′ sequence covering the 17 bp palindromic sequence (−8 to +8) conserved in promoters of the *fap* regulon. The non-crystallographic two-fold axis relating the two FapR homodimers in the crystal structure is indicated by the green arrow. (**C**) Schematic view of the DNA conformation within the FapR–DNA complex. Each base pair is represented by a single block, and the dark-shaded side indicates the minor groove. The actual base-step parameters are reported in Table S2.

The two DBDs from the asymmetric homodimer interact in a similar manner with DNA. The recognition helix α3 from the helix-turn-helix motif penetrates the double-helix major groove and makes extensive interactions with the two DNA chains (Fig. 2A). The *Sa*FapR-DNA interface mainly involves the DNA backbone phosphates (Fig. 2B), except for base-specific interactions of Gln41 (from the recognition helix α3) in the major groove and Arg56 (from the linker helix α_L_) in the minor groove. Importantly, the insertion of the guanidinium groups of this arginine from both protomers promotes the opening of the minor groove, inducing a pronounced local bending of DNA (Fig. 2C). The two phosphate-contacting residues from helix α1 (Lys10 and Arg13), as well as residues from the recognition helix α3 and the C-terminal half of the linker helix α_L_ (which include most other DNA-contacting residues) are highly conserved in the entire FapR protein family (Fig. S6), indicating a conserved mode of DNA-binding. The N-terminus of the protein (preceding helix α1) faces the adjacent minor groove in the crystal structure (Fig. 2A), suggesting that it could be engaged in additional DNA interactions. However, this region is poorly conserved in the FapR protein family and is disordered in the crystal structure.

### Structure of the repressor-effector complex

**Figure 3:**
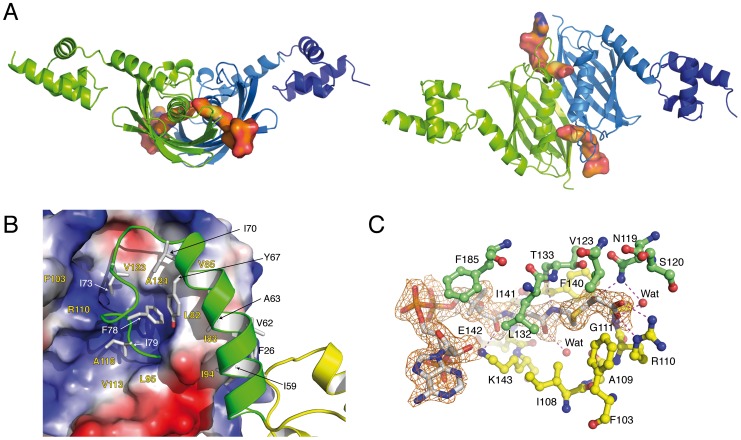
Overall structures of the malonyl-CoA-bound forms of *Sa*FapR. (**A**) Cartoon showing the structure of the *Sa*FapR-malonyl-CoA homodimer in two different views. The first protomer is shown in green; the second protomer is shown in blue (the helix-turn-helix motif - in dark blue - was partially visible in the electron density map but was not included in the final model due to high protein mobility). Bound malonyl-CoA is shown in surface representation. (**B**) Closer view of the interactions between the central hot-dog fold (electrostatic surface representation) and the linker helix (green). Hydrophobic side chains involved in inter-domain interactions are labeled. (**C**) Electron density map of malonyl-CoA and protein-ligand interactions. Hydrogen bonds are indicated by dashed lines and protein residues from each protomer are colored green and yellow respectively.

The crystal structure of full-length *Sa*FapR in complex with malonyl-CoA, determined at 1.9 Å resolution (Table 1), revealed a different, more compact conformation of the repressor. Most notably, the two amphipatic linker helices α_L_, instead of interacting with each other as in the DNA-bound repressor, now bind to either side of the central dimeric EBD (Fig. 3A). This results in a protein conformation with the two helix-turn-helix domains far apart from each other, corresponding to an incompetent DNA-binding state. Interactions of helix α_L_ with the lateral face of the EBD play a major role in stabilizing the observed quaternary organization of the protein, mainly through extended hydrophobic contacts between aliphatic and aromatic side chains (Fig. 3B). In addition, several hydrogen bonding and salt bridge interactions (Fig. S5B) lock a well-defined structure for the loop connecting helix α_L_ to the first β-strand of the EBD (residues 70–82), which further restrict the mobility of α_L_ and contrasts with the loose asymmetric conformation observed for this loop in the DNA-bound *Sa*FapR homodimer (Fig. 1A).

The phosphopantetheine and malonyl groups of malonyl-CoA are well defined in the electron density map (Fig. 3C). The phosphopantetheine group binds within a tunnel at the interface between the two protomers in the *Sa*FapR homodimer, and adopts the same conformation as observed in many acyl-CoA-binding proteins displaying the hot-dog fold [20], [24]. This binding mode results in the complete occlusion of the ligand malonate from the bulk solvent. The charged carboxylate group of malonate is neutralized through a salt bridge with the guanidinium side-chain of Arg110, and makes additional hydrogen bonding interactions with the main-chain nitrogen from Gly111 and the side-chain of Asn119′ from the second protomer (Fig. 3C). The engagement of Arg110 in malonyl-CoA-binding triggers a local reorganization of hydrogen-bonding interactions (Fig. S5B) and surface reshaping that further stabilize the loop connecting α_L_ to the first β-strand of the hot-dog fold in the non-productive conformation (Fig. 3A), thus preventing DNA binding. On the other hand, the adenosine 3′-phosphate moiety of malonyl-CoA is largely exposed to the solvent (it is partially disordered in one of the two protomers) and makes no specific contacts with the protein. This implies that *Sa*FapR specifically recognizes the malonyl-phosphopantetheine moiety of the ligand, in agreement with the observation that either malonyl-CoA or malonyl-acyl carrier protein (malonyl-ACP) can indistinctly function as effector molecules [28].

A detailed comparison of the malonyl-CoA complexes between full-length *Sa*FapR and the truncated form of *Bs*FapR (lacking the DBDs) revealed a conserved structural arrangement of the EBD core, and ligand binding promoted the same conformation of the connecting loop α_L_-β1 (Fig. S7). On the other hand, helix α_L_ displays a different organization, due in part to the absence of the DBDs. This helix protrudes away from the EBD core in *Bs*FapR to get involved in crystal packing contacts [20]. Interestingly, hydrophobic residues engaged in these inter-domain interactions are largely invariant in the whole FapR family (Fig. S6), as are also key residues involved in electrostatic interactions (Fig. S5). Altogether, the structural alignment indicates not only an identical mode of malonyl-CoA binding but also the conservation of the DBD – α_L_ – EBD interactions required to stabilize the FapR-malonyl-CoA complex as visualized in the *Sa*FapR model.

### The overall structure of *Sa*FapR

**Figure 4:**
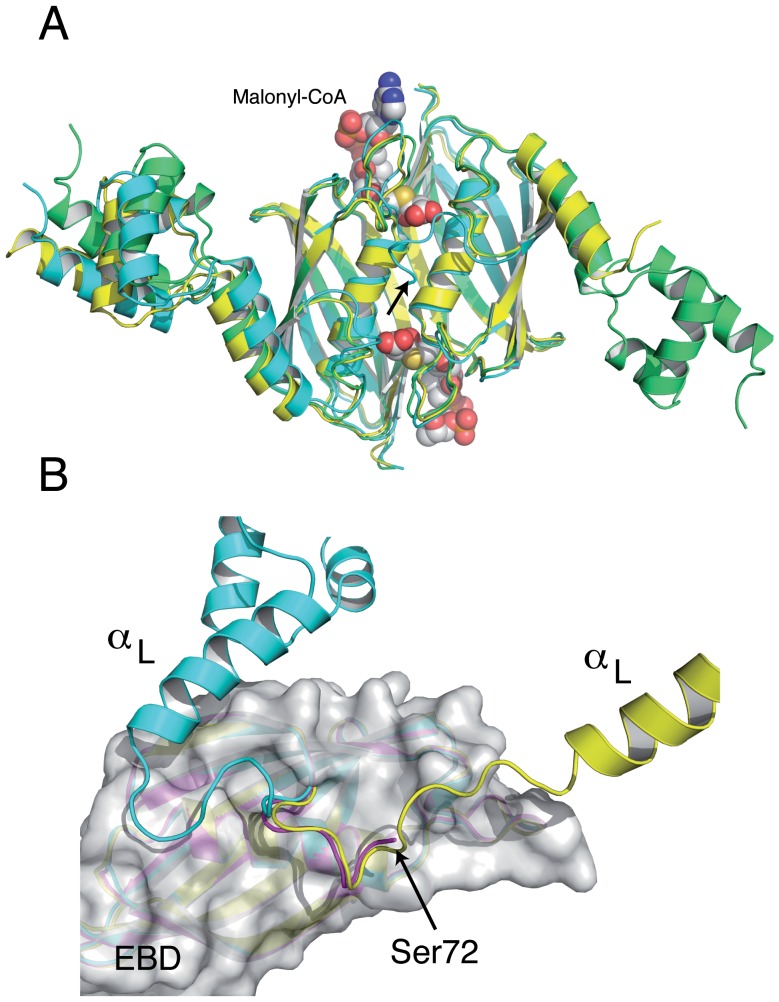
The structures of the *Sa*FapR homodimer in the absence of ligands display distinctive features of either the malonyl-CoA-bound or the DNA-bound forms of the repressor. (**A**) Structural superposition of the ligand-free repressor in two different crystal forms (green and cyan) with the malonyl-CoA-bound form (yellow), revealing a similar quaternary organization. Bound malonyl-CoA is shown as solid spheres. Note that helix α_L_ and its attached DBD are flexible (not modeled) in one monomer of the ligand-free repressor (in cyan, at right). (**B**) Superposition of this same monomer (magenta) with an equivalent subunit from the malonyl-CoA-bound (cyan) and the DNA-bound (yellow) forms of the repressor. The EBD region (grey molecular surface) is identical for all three monomers. The loop connecting helix α_L_ with the first β-strand of the EBD in the ligand-free subunit (residues 72–76) has the same conformation as observed in the DNA-bound structure. In both panels, the arrow indicates the first visible residue (Ser72) of the subunit with a disordered helix α_L_.

The crystal structure of full-length *Sa*FapR in the absence of ligands has been obtained in two different crystal forms (Table 1). Interestingly, in three out of four crystallographically independent protomers the repressor has the same non-productive quaternary arrangement as observed in the structure of malonyl-CoA-bound *Sa*FapR, with helix α_L_ bound to the lateral face of the EBD (Fig. 4A), strongly suggesting that this compact structure likely represents a conformation of the protein in solution. However, the helix-turn-helix motifs display high temperature factors or even partial disorder, and are engaged in extensive crystal contacts, suggesting the coexistence of alternative conformational states in solution characterized by flexible DBDs. In that sense, in one protomer of the crystal form 2 (Table 1) both helix α_L_ and its associated DBD are highly flexible and could not be modeled, suggesting a marginal stability of the observed quaternary arrangement. Moreover, the first visible residues of this same monomer (positions 72–77, connecting helix α_L_ with the first β-strand of the EBD) adopt a conformation that differs from that observed in the other ligand-free or malonyl-CoA-bound protomers, but resembles that found for one subunit of the asymmetric DNA-bound form of the repressor (Fig. 4B).

### Ligand-induced structural transition

**Figure 5:**
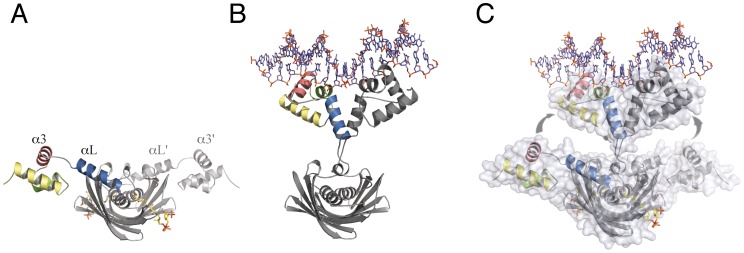
The transitional switch between the relaxed and tense states of the repressor involves a significant rearrangement of the DBDs. (**A**) *Sa*FapR in complex with malonyl-CoA (shown in stick representation), tense state. (**B**) *Sa*FapR in complex with DNA, relaxed state in which the amphipathic helix α_L_ from each protomer associates with each other. (**C**) Superposition of the two conformational states of the repressor illustrating the structural transition. Solvent accessible surfaces are shown in transparent to highlight the DNA-induced dissociation of the invariant effector-binding domain from the DBDs. The molecules are shown in light (relaxed) and dark (tense) grey, except for the helices from one DBD (colored).

The structure of *Sa*FapR in complex with the DNA operator revealed a strikingly different overall conformation of the protein, compared to those of the ligand-free and the effector-bound repressor. As highlighted by the crystal structures described above, the transitional switch between the relaxed (DNA-bound) and tense (malonyl-CoA-bound) forms of the repressor involves a large-scale structural rearrangement (Fig. 5). The amphipathic helix α_L_, whose hydrophobic face binds to the protein core in the tense state (Fig. 5A), structurally dissociates and moves >30 Å to interact with the same helix from the other protomer and with DNA in the relaxed state (Fig. 5B). This DNA-driven process requires partial unwinding of the linker helix α_L_ and the solvent-exposure of hydrophobic side-chains from the EBD (Leu82, Ile83, Val85, Ile94, Val123) and from the loop immediately following helix α_L_ (Ile70, Phe78) in both protomers. Such a substantial conformational rearrangement between the tense and relaxed states (Fig. 5C) contrasts with the more subtle structural changes observed for other well-studied bacterial classes of allosteric transcriptional regulators such as the tetracycline [29] or lactose [30] repressors, illustrating that specific physiological responses can be achieved by a variety of mechanisms.

### Disruption of membrane lipid homeostasis is lethal for *S. aureus*

**Figure 6:**
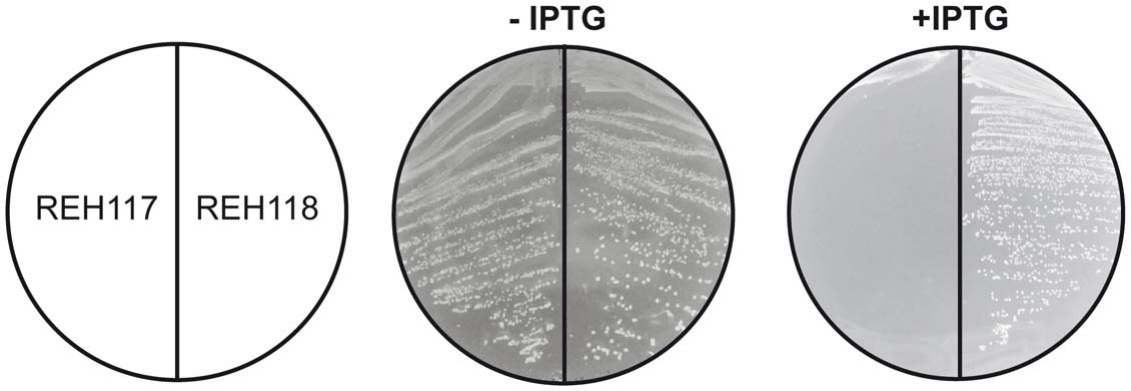
Expression of the *Sa*FapR_G11V, L132W_ superrepresor is lethal for *S. aureus* and fatty acid supplementation cannot overcome growth inhibition. The figure shows the growth of strain RN4220ΔfapR expressing *Sa*FapR_G11V, L132W_ (REH117) or *Sa*FapR_WT_ (REH118) under the tight inducible P*spacOid* promoter on THA plates in the absence or presence of 10 mM IPTG. Identical results were obtained for strain REH117 growing in 10 mM IPTG when supplemented with either Tween80 (0.1%) or 500 µM of the following fatty acids: palmitic acid (16∶0), oleic acid (18∶1), 16∶0+18∶1, anteiso 17∶0 (a17∶0), a15∶0+a17∶0, or 18∶0+a17∶0.

Bacterial FASII has been identified as a promising target for antibacterial drug discovery. Nevertheless, Brinster et al [31] have questioned the feasibility of this approach in Gram-positive pathogens based on the finding that FASII is not essential in *Streptococcus agalactiae* if the bacterium is supplemented with fatty acids or human serum. This controversy was recently clarified by Parsons et al [32], who showed that externally added fatty acids downregulate the activity of the acetyl-CoA carboxylase in some Gram-positive pathogens, such as *Streptococcus pneumoniae*. This biochemical mechanism suppresses the malonyl-CoA levels allowing fatty acid supplements to replace endogenous fatty acids completely, thus rescuing bacterial FASII inhibition. In *S. aureus*, this feed-back regulatory mechanism is not present [32] and thus external fatty acids do not circumvent the treatment of this bacterium with FASII inhibitors. These results and the regulatory properties of the *acc* genes led the authors to propose that pathogens containing FapR would likely be sensitive to FASII inhibitors, regardless of the addition of external fatty acids [32]. These considerations prompted us to test if structure-based mutations predicted to disrupt the *Sa*FapR-malonyl-CoA interaction are lethal for *S. aureus* even in the presence of extracellular fatty acids. To this end, based on the structural information and on our previous work [20], we substituted Arg110 by alanine to disrupt the key interaction with the malonyl carboxylate, and introduced a double substitution (Gly111Val, Leu132Trp) to block the ligand-binding tunnel that accommodates the phosphopantetheine moiety in the repressor-effector complex (Fig. 3C). These mutants were expected to retain their DNA-binding capacity and to permanently repress the expression of the *fap* regulon, independently of the metabolic conditions. To test this prediction we engineered *S. aureus fapR* null mutants to produce the protein variants in response to an inducer (IPTG). IPTG-induced expression of *Sa*FapR_R110A_ caused a small drop in cell viability (data not shown), while cells expressing *Sa*FapR_G111V,L132W_ failed to grow (Fig. 6). A variety of fatty acids, including oleic acid, a common mammalian fatty acid, and anteiso saturated fatty acids, the most abundant acyl chains found in *S. aureus* phospholipids were unable to overcome the growth inhibition caused by the expression of the superrepressor *Sa*FapR_G111V,L132W_ in *S. aureus* (Fig. 6). These results clearly show that disruption of the membrane lipid homeostasis mimics the effects of FASII inhibitors in *S. aureus* strongly supporting the notion that exogenous fatty acids cannot replace the endogenously produced acyl chain in this bacterium [32].

## Discussion

The structures of *Sa*FapR, in three relevant states of its regulation cycle, uncover a complex biological switch, characterized by completely different protein-protein interactions involving in all cases the linker helix α_L_ and so leading to a distinct quaternary arrangement_._for the tense and relaxed states of the repressor (Fig. 5). Similarly to other homodimeric proteins studied by single-molecule or NMR relaxation approaches [33], [34], our crystallographic studies suggest that the two conformational states of *Sa*FapR (i.e. with EBD-bound or EBD-detached DBDs) can be populated in the ligand-free repressor species. Thus, a higher cellular concentration of malonyl-CoA would not only trigger the conformational changes that disrupt the *Sa*FapR-operator complex [20], but would also promote the dynamic shift of the ligand-free repressor population towards the tense state.

Our results highlight the ability of FapR to monitor the levels of malonyl-CoA and appropriately tune gene expression to control lipid metabolism, ensuring that the phospholipid biosynthetic pathway will be supplied with appropriate levels of fatty acids either synthesized endogenously or incorporated from the environment. Bacterial FASII is a target actively pursued by several research groups to control bacterial pathogens [35]. A controversy surrounding FASII as a suitable antibiotic target for *S. aureus* was based on the ability of this pathogen to incorporate extracellular fatty acids [36]. This apparent discrepancy was recently clarified by showing that although exogenous fatty acids indeed are incorporated by *S. aureus*, following its conversion to acyl-ACP, they cannot deplete the generation of malonyl-CoA and malonyl-ACP [32]. Thus, these fatty acid intermediates release FapR from its binding sites [28] and are used by FASII to initiate new acyl chains. Thus, when a FASII inhibitor is deployed against *S. aureus*, the initiation of new acyl chains continues leading to depletion of ACP, which is correlated with diminished exogenous fatty acids incorporation into phospholipids [32]. Strikingly, *Sa*FapR, not only control the expression of the FASII pathway, but also regulate the expression of key enzymes required for phospholipid synthesis such as PlsX and PlsC [18], [37]. Most of the Gram-positive pathogens rely on the PlsX/PlsY system to initiate phospholipid synthesis by converting acyl-ACP to acyl-P0_4_ by PlsX followed by the transfer of the fatty acid to the 1 position of glycerol-PO_4_ by the PlsY acyltransferase [37], [38]. A strength of targeting these steps in lipid synthesis is that acyltransferases inhibition cannot be circumvented by supplementation with extracellular fatty acids. Thus, targeting of lipid synthesis with compounds that block the expression of PlsX in *S. aureus* cannot be ignored, specially taking into account that it has been reported that extracellular fatty acids increase the MIC for FASII inhibitors in this important pathogen. [32], [39]. In this sense, the unique mode of action of FapR and our encouraging *in vivo* results validate lipid homeostasis as a promising target for new antibacterial drug discovery in Gram-positive bacteria.

Conserved in many Gram-positive bacteria, FapR is a paradigm of a feed-forward-controlled lipid transcriptional regulator. In other characterized bacterial lipid regulators it is the long acyl-chain end products of the FASII pathway that act as feedback ligands of lipid transcriptional regulators [40]. The effector-binding domains of these proteins, such as *E. coli* FadR [12] or the TetR-like *P. aeruginosa* DesT [17], frequently display an α-helical structure with a loose specificity for long-chain acyl-CoA molecules, possibly because the permissive nature of helix-helix interactions provide a suitable platform to evolve a binding site for fatty acid molecules of varying lengths. In contrast, a high effector-binding specificity is required for the feed-forward regulation mechanism of the FapR repressor family [19], which entails the recognition of an upstream biosynthetic intermediate, namely the product of the first committed step in fatty acid biosynthesis. This high specificity is achieved in *Sa*FapR by caging the charged malonyl group inside a relatively stiff internal binding pocket, and may be the reason why the hot-dog fold was recruited for this function. Nevertheless, it could be expected that, in organisms using the FapR pathway, a complementary feed-back regulatory loop should also operate at a biochemical level, for instance by controlling the synthesis of malonyl-CoA [37]. This would imply that lipid homeostasis in FapR-containing bacteria would be exquisitely regulated by feed-back and feed-forward mechanisms, as it is indeed the case in higher organisms ranging from *Caenorhabditis elegans* to humans [41].

## Materials and Methods

### *In vivo* studies

In frame *fapR* deletion mutants of *S. aureus* strains RN4220 and HG001 [42], RN4220_Δ*fapR*_ and HG001_Δ*fapR*_ respectively, were constructed and the expression level of the *plsX* and *fabH* genes, belonging to the *fap* regulon, was analyzed by real time PCR as described in Text S1. To evaluate the activity of the *S. aureus* repressor in *B. subtilis*, we used the Δ*fapR* mutant strain GS416 that contains a P*fabHB*-*lacZ* reporter fusion and expressed *S. aureus fapR* from the xylose inducible P*xylA* promoter (for further details see Text S1).

Mutants *Sa*FapR_R110A_ and *Sa*FapR_G111V,L132W_, impaired in malonyl-CoA binding were obtained by site-directed mutagenesis using *S. aureus* RN4220 genomic DNA as template, mutagenic oligonucleotides and overlap-extension PCR. The replicative vector pOS1 [43] and the tight IPTG-regulated P*spacOid* promoter from pMUTIN4 were used for ectopical expression of *fapR* alleles in RN4220_Δ*fapR*_ (see Text S1). *S. aureus* transformants were selected in THA plates supplemented with cloramphenicol (5 µg/ml) and expression of *fapR* alleles was achieved by addition of 10 mM IPTG. To evaluate the effect of an external source of fatty acids THA plates were supplemented with 500 µM of the stated fatty acids or 0.1% Tween80 (Fig. 6).

### Protein expression and purification

The *S. aureus fapR* gene was cloned into the pET15b vector (Novagen) as described in Text S1 and expressed in *E. coli* BL21/pLysS. Bacterial cultures were grown in LB supplemented with ampiciline 100 µg/ml and chloramphenicol 10 µg/ml at 37°C until OD_600_ 0.6. Expression was induced with 0.5 mM IPTG at 20°C for 17 hours. Cells were harvested by centrifugation at 4°C, and protein-containing fractions from a Ni^2+^-affinity chromatography on a HisTrap column (GE Healthcare) were dialyzed overnight against an excess volume of 50 mM Tris-HCl pH 7.6, 300 mM NaCl and 1 mM DTT at room temperature, in the presence of a 1/40 (w/w) ratio of His-tagged TEV protease. After dialysis, a second affinity chromatography step was performed in the presence of 20 mM imidazole, the flow through was concentrated and injected into a HiLoad 16/60 Superdex 75 prep grade column (GE Healthcare) equilibrated with 50 mM Tris-HCl, pH 7.6, and 300 mM NaCl. After gel filtration *Sa*FapR was dialyzed at room temperature against 20 mM Tris-HCl pH 7.6 and 50 mM NaCl, concentrated to 15 mg/ml and stored in aliquots at −80°C for further use. Selenomethionine (SeMet)-labeled *Sa*FapR was obtained using the *E. coli* strain B834 (Novagen) grown in M9 medium containing 0.2 g/l selenomethionine and purified as above.

### Crystallographic studies

Crystals of the repressor and its complexes with the effector and operator molecules were obtained as described in Text S1. All diffraction datasets were collected from single crystals at 100 K using synchrotron radiation at beamlines ID14.4 and ID29 (European Synchrotron Radiation Facility, Grenoble, France) or Proxima 1 (SOLEIL, Saint-Aubin, France). Data were processed with either XDS [44] or iMosflm [45] and scaled with XSCALE from the XDS package or SCALA from the CCP4 suite [46]. The crystal structures of *Sa*FapR alone and its complex with malonyl-CoA were solved by molecular replacement methods using the program AMoRe [47] and the effector-binding domain of *B. subtilis* FapR (PDB entry 2F41) as the search model. In all cases, the missing DNA-binding domains were manually traced from sigma A-weighted Fourier difference maps. The structure of the repressor-operator complex was solved by a combination of molecular replacement and single-wavelength anomalous diffraction (SAD) techniques using SeMet-labeled *Sa*FapR. The selenium substructure was determined with the program SHELXD [48] and further refined with the program SHARP [49]. Structures were refined with REFMAC5 [50] or BUSTER [51] (for the SeMet-labeled protein-DNA complex) using a TLS model and non-crystallographic symmetry restraints when present, alternated with manual rebuilding with COOT [52]. Models were validated through the MolProbity server [53]. Data collection and refinement statistics are reported in Table 1. Graphic figures were generated and rendered with programs Pymol [54] and 3DNA [55].

### Isothermal titration calorimetry

ITC experiments were performed using the high precision VP-ITC system (MicroCal Inc., MA) and quantified with the Origin7 software provided by the manufacturer. All molecules were dissolved in 50 mM TrisHCl, pH 8, 150 mM NaCl and the binding enthalpies were measured by injecting the *Sa*FapR solution into the calorimetric cell containing the 40 bp DNA solution. Heat signals were corrected for the heats of dilution and normalized to the amount of compound injected. Complementary DNA strands were heated to 90°C and annealed by a stepwise decrease to 25°C followed by 30 min on ice prior to use; DNA concentration was determined by absorption at 260 nm.

### Accession codes

Crystallographic coordinates and structure factors were deposited in the Protein Data Bank, with accession codes 4a0x, 4a0y, 4a0z and 4a12.

## Supporting Information

Figure S1 Fatty acid synthesis and phospholipid initiation steps in *Bacillus subtilis*. The FabH condensing enzymes initiates the cycles of fatty acid elongation by condensation of acyl-CoA primers with malonyl-ACP (3a). The resultant β-ketoester is reduced by the β-ketoacyl-ACP reductase (4). Then, the β-hydroxyacyl-ACP is dehydrated to the *trans*-2 unsaturated acyl-ACP by β-hydroxyacyl-ACP dehydrase (5), which is finally reduced by enoyl reductase (6). Subsequent rounds of elongation are initiated by the elongation-condensing enzyme FabF (3b) to generate an acyl-ACP two carbons longer than the original acyl-ACP at the end of each cycle. The long chain acyl-ACP end products of fatty acid synthesis are transacylated in three steps to glycerolphosphate, to generate phosphatidic acid (PA), a key intermediate in the synthesis of phospholipids. First, PlsX catalyzes the synthesis of fatty acylphosphate from acyl-ACP (7); then, PlsY transfers the fatty acid from the activated acyl intermediate to the 1-position of glycerol-3P (8) and finally, lyso-PA is acylated to PA by PlsC (9). Malonyl-CoA is generated from acetyl-CoA by acetyl-CoA carboxylase (1) and then is transferred to ACP by malonyl-CoA transacylase (2). Expression of the genes surrounded by shaded ellipses is repressed by FapR, whose activity is, in turn, antagonized by malonyl-CoA (enclosed in a rectangular box). R denotes the terminal group of branched-chain or straight-chain fatty acids.
(TIF)

Figure S2 Effect of malonyl-CoA and short-chain acyl-CoA thioesters on binding of *Sa*FapR to P*fapR*. (**A**) Electrophoretic shift assay showing the binding of *Sa*FapR to P*fapR*. Increasing amounts of *Sa*FapR were incubated with 30 nM of double strand P*fapR* probe (left panel) or non-specific DNA (right panel). (**B**) Effect of malonyl-CoA on *Sa*FapR binding to P*fapR*. The gel mobility shift assay was performed with two concentrations of *Sa*FapR (100 nM or 500 nM), previously incubated at different concentrations of malonyl-CoA for 15 min at room temperature followed by addition of the probe to the *Sa*FapR-malonyl-CoA complex. (**c**) Effect of malonyl-CoA analogs on *Sa*FapR binding to P*fapR. Sa*FapR (200 nM) was previously incubated, as described in b, with malonyl-CoA (lanes 3 and 4), acetyl-CoA (lanes 5 and 6), propionyl-CoA (lanes 7 and 8) or succinyl-CoA (lanes 9 and 10) before adding the P*fapR* probe to the reaction mixture. Gels were stained with SYBR GREEN and scanned at 530 nm.
(TIF)

Figure S3 Conservation of FapR function in *S. aureus*. (**A**) RT-PCR results showing the expression of two genes from the *fap* regulon (*plsX*, *fabH*) in two different strains (RN4220, HG001). Relative levels of transcripts were measured by qRT-PCR. Expression levels were normalized using 16S RNA as an internal standard and are indicated as an n-fold change, expressed as the means and standard deviations of quadruplicate experiments. (**B**) *Sa*FapR complements a *B. subtilis fapR* null strain. Cultures of strains GS413 (squares; Δ*fapR*, P*fabHB-lacZ*, *amyE*::*spc*), GS373 (circles; Δ*fapR*, P*fabHB-lacZ*, *amyE*::P*xyl-BsfapR*:*spc*) and GS416 (triangles; Δ*fapR*, P*fabHB-lacZ*, *amyE*::P*xyl-SafapR*:*spc*) were incubated in LB medium in the presence of xylose 0.1% (w/v) at 37°C and growth was monitored. Samples were removed to assay ß-galactosidase-specific activity at the indicated times. A single experiment representative of three repeats is shown. Solid lines, ß-galactosidase-specific activity; dotted lines, OD_525_.
(TIF)

Figure S4 Alignment of the promoter regions of the genes and operons regulated by FapR. The -10 motifs of RNA polymerase binding boxes are indicated with rectangles. The conserved inverted repeat sequence is shaded and a consensus sequence is indicated at the bottom line. Bases are numbered related to the first base of the translation initiation codon.
(TIF)

Figure S5 Different roles of the amphipatic linker helix α_L_ in stabilizing the *Sa*FapR-DNA and *Sa*FapR-malonyl-CoA complexes. (**A**) Close view of the *Sa*FapR-DNA complex. (**B**) Close view of the interactions between the EBD, the linker helix (green) and the DBD in the structure of the *Sa*FapR-malonyl-CoA complex. In both cases, hydrophobic and hydrophilic side chains involved in inter-domain interactions are labeled. Malonyl-CoA binding triggers significant conformational changes in three main regions of the repressor: the connecting loop α_L_ - β1, the amphipathic helix α_L_ and the DBD. The side-chain of Arg110 from the EBD makes a key hydrogen bond with the main-chain carbonyl group of Glu77′ in the loop. In addition, the Glu77′ side-chain is hydrogen bonded to the main chain of His104 from the EBD, facilitating the formation of a strong salt bridge between the side-chains of Lys105 and Glu74′. The Ser102 side-chain makes a strong hydrogen bond with Val123, whereas its main chain interacts through hydrogen bonding networks with Ser72, Pro129 and a water molecule. Furthermore, the main-chain of Gln69 makes an electrostatic interaction with the side-chain of Lys127. These newly formed electrostatic/hydrogen-bond interactions allow the connecting loop α_L_ - β1 to firmly interact with the core of the EBD. As a consequence, the amphipathic helix α_L_ forms now an extended hydrophobic interface (Leu55, Ile59 and Val62), with both β1 (Ile83) and β2 (Ile94) strands of the EBD, and the α1–α2 connecting loop of the DBD (Pro25, Phe26 and Thr28). This structural arrangement is further stabilized by key electrostatic interactions. Arg56 makes a salt bridge with Asp96 located at the top of β2 and the side-chain of Asn66 makes a hydrogen bond with the carbonyl group of the main-chain of Leu82 on the β1 strand. Moreover, the side-chain of Tyr67 is oriented towards a hydrophobic pocket including residues Leu82, Val85 from β1, and Ile70, Ile73 and Phe78 from the connecting loop α_L_ - β1.
(TIF)

Figure S6 Sequence alignment of FapR. Amino acid sequence and secondary structure elements (green for DBD, pink for EBD) of *Sa*FapR. Residues that interact with the pantetheine group of malonyl-CoA and with DNA are underlined by open and closed circles, respectively. Wavy lines indicate disordered regions in the crystal structure. Colored amino acid positions indicate highly conserved residues in non-redundant FapR sequences from 67 different bacterial species (yellow, >80%; orange, >95%).
(TIF)

Figure S7 Structural superposition of the *Bs*FapRΔ43-malonyl-CoA and *Sa*FapR-malonyl-CoA complexes. Although the orientation of helix α_L_ is different (it is involved in crystal packing contacts in *Bs*FapRΔ43), binding of malonyl-CoA promotes the same conformation of the loop connecting this helix with the first β-strand of the EBD.
(TIF)

Table S1 The *fap* regulon in *B. subtilis* and *S. aureus*.
(DOC)

Table S2 Local base-pair step parameters of the DNA in complex with *Sa*FapR.
(DOC)

Table S3 Oligonucleotides used in this work.
(DOC)

Text S1 Supplemental information, including additional experimental procedures.
(DOC)
