# Supplementary figures and images for: Structural Basis for Feed-Forward Transcriptional Regulation of Membrane Lipid Homeostasis in Staphylococcus aureus
Source: PLoS Pathog. 2013 Jan 3;9(1):e1003108. doi: 10.1371/journal.ppat.1003108 (PMC3536700; doi:10.1371/journal.ppat.1003108)

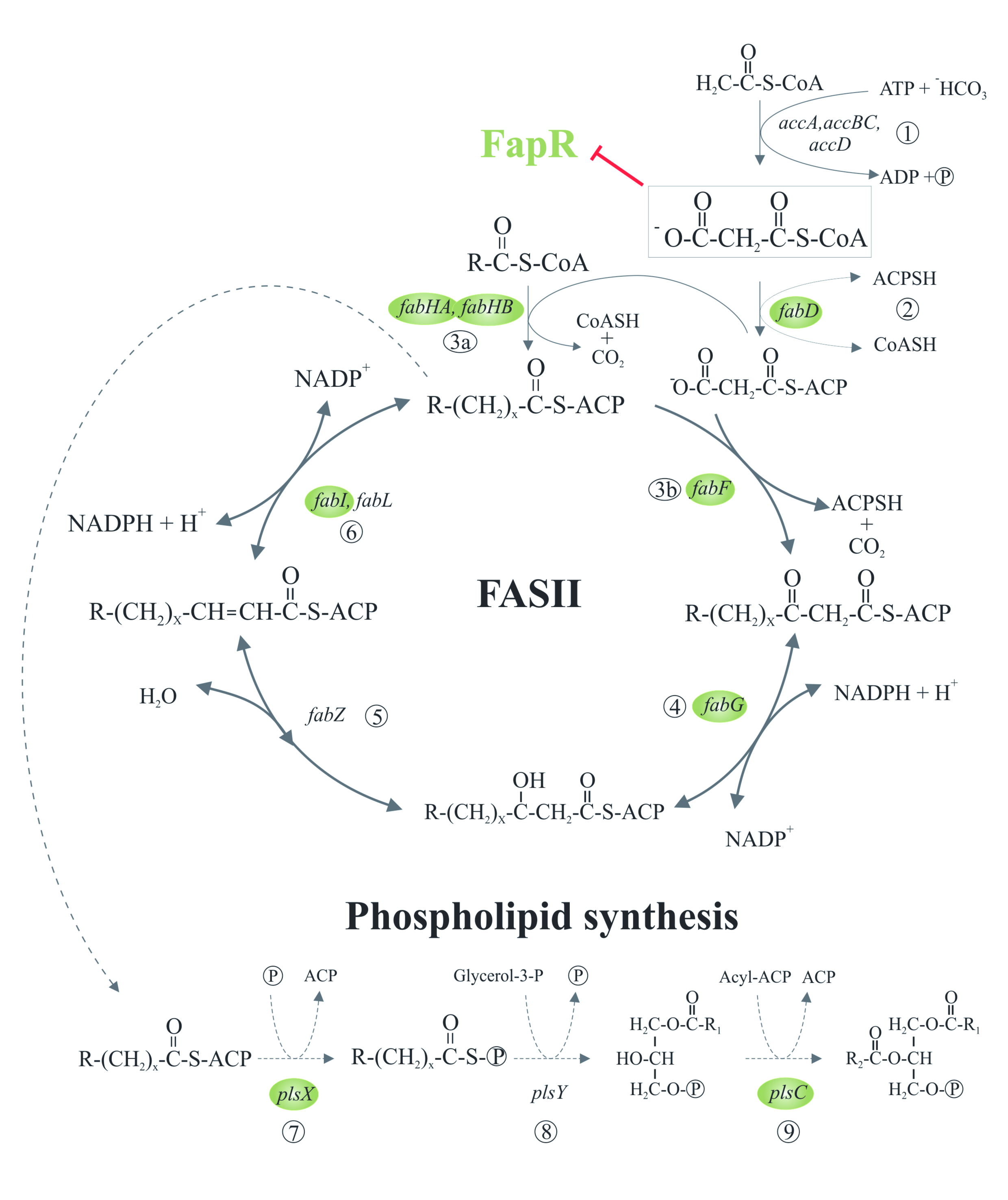

Supplement: Figure S1 — Fatty acid synthesis and phospholipid initiation steps in Bacillus subtilis. The FabH condensing enzymes initiates the cycles of fatty acid elongation by condensation of acyl-CoA primers with malonyl-ACP (3a). The resultant β-ketoester is reduced by the β-ketoacyl-ACP reductase (4). Then, the β-hydroxyacyl-ACP is dehydrated to the trans-2 unsaturated acyl-ACP by β-hydroxyacyl-ACP dehydrase (5), which is finally reduced by enoyl reductase (6). Subsequent rounds of elongation are initiated by the elongation-condensing enzyme FabF (3b) to generate an acyl-ACP two carbons longer than the original acyl-ACP at the end of each cycle. The long chain acyl-ACP end products of fatty acid synthesis are transacylated in three steps to glycerolphosphate, to generate phosphatidic acid (PA), a key intermediate in the synthesis of phospholipids. First, PlsX catalyzes the synthesis of fatty acylphosphate from acyl-ACP (7); then, PlsY transfers the fatty acid from the activated acyl intermediate to the 1-position of glycerol-3P (8) and finally, lyso-PA is acylated to PA by PlsC (9). Malonyl-CoA is generated from acetyl-CoA by acetyl-CoA carboxylase (1) and then is transferred to ACP by malonyl-CoA transacylase (2). Expression of the genes surrounded by shaded ellipses is repressed by FapR, whose activity is, in turn, antagonized by malonyl-CoA (enclosed in a rectangular box). R denotes the terminal group of branched-chain or straight-chain fatty acids. (TIF) [file ppat.1003108.s001.tif]

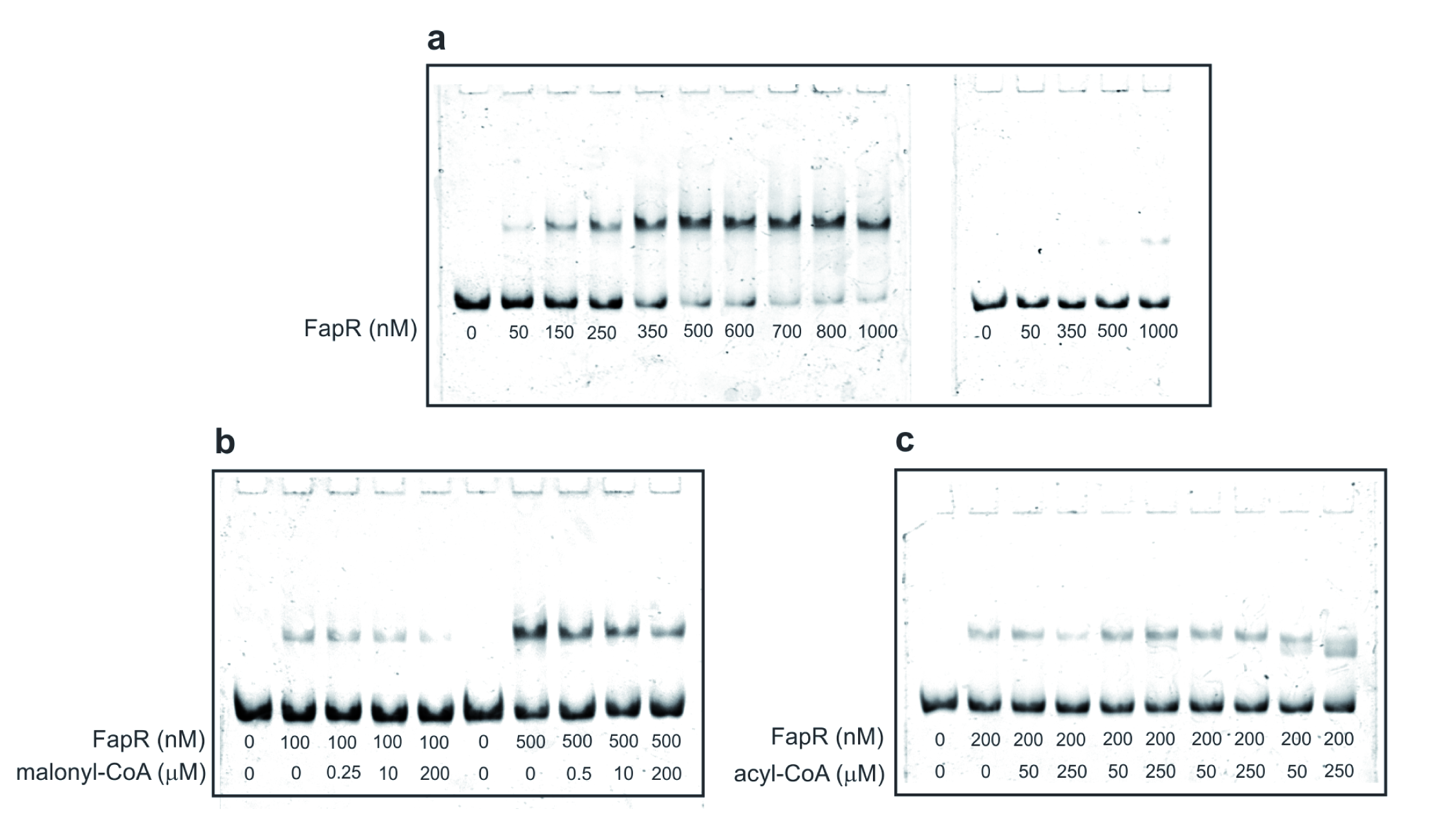

Supplement: Figure S2 — Effect of malonyl-CoA and short-chain acyl-CoA thioesters on binding of SaFapR to PfapR. (A) Electrophoretic shift assay showing the binding of SaFapR to PfapR. Increasing amounts of SaFapR were incubated with 30 nM of double strand PfapR probe (left panel) or non-specific DNA (right panel). (B) Effect of malonyl-CoA on SaFapR binding to PfapR. The gel mobility shift assay was performed with two concentrations of SaFapR (100 nM or 500 nM), previously incubated at different concentrations of malonyl-CoA for 15 min at room temperature followed by addition of the probe to the SaFapR-malonyl-CoA complex. (c) Effect of malonyl-CoA analogs on SaFapR binding to PfapR. SaFapR (200 nM) was previously incubated, as described in b, with malonyl-CoA (lanes 3 and 4), acetyl-CoA (lanes 5 and 6), propionyl-CoA (lanes 7 and 8) or succinyl-CoA (lanes 9 and 10) before adding the PfapR probe to the reaction mixture. Gels were stained with SYBR GREEN and scanned at 530 nm. (TIF) [file ppat.1003108.s002.tif]

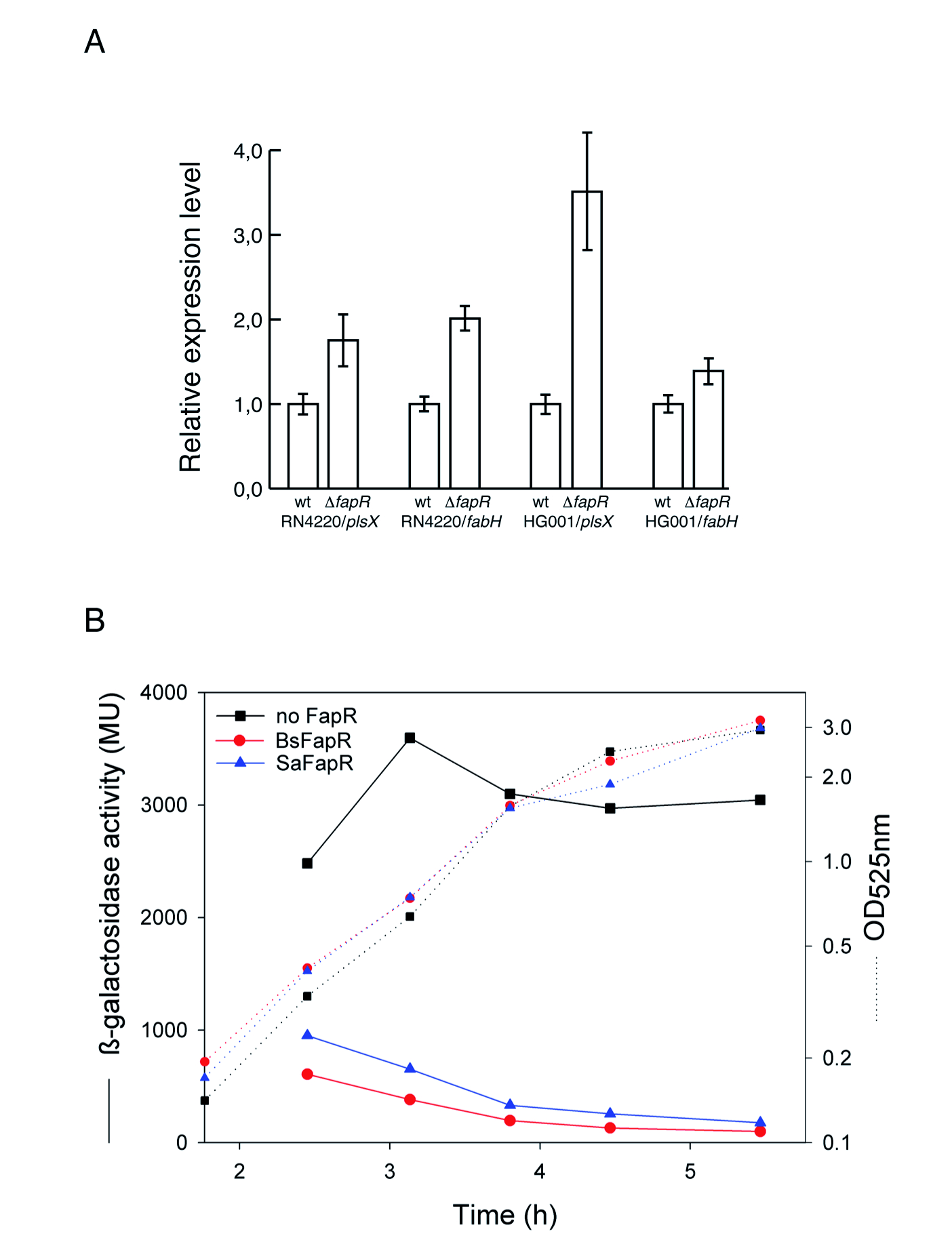

Supplement: Figure S3 — Conservation of FapR function in S. aureus. (A) RT-PCR results showing the expression of two genes from the fap regulon (plsX, fabH) in two different strains (RN4220, HG001). Relative levels of transcripts were measured by qRT-PCR. Expression levels were normalized using 16S RNA as an internal standard and are indicated as an n-fold change, expressed as the means and standard deviations of quadruplicate experiments. (B) SaFapR complements a B. subtilis fapR null strain. Cultures of strains GS413 (squares; ΔfapR, PfabHB-lacZ, amyE::spc), GS373 (circles; ΔfapR, PfabHB-lacZ, amyE::Pxyl-BsfapR:spc) and GS416 (triangles; ΔfapR, PfabHB-lacZ, amyE::Pxyl-SafapR:spc) were incubated in LB medium in the presence of xylose 0.1% (w/v) at 37°C and growth was monitored. Samples were removed to assay ß-galactosidase-specific activity at the indicated times. A single experiment representative of three repeats is shown. Solid lines, ß-galactosidase-specific activity; dotted lines, OD525. (TIF) [file ppat.1003108.s003.tif]

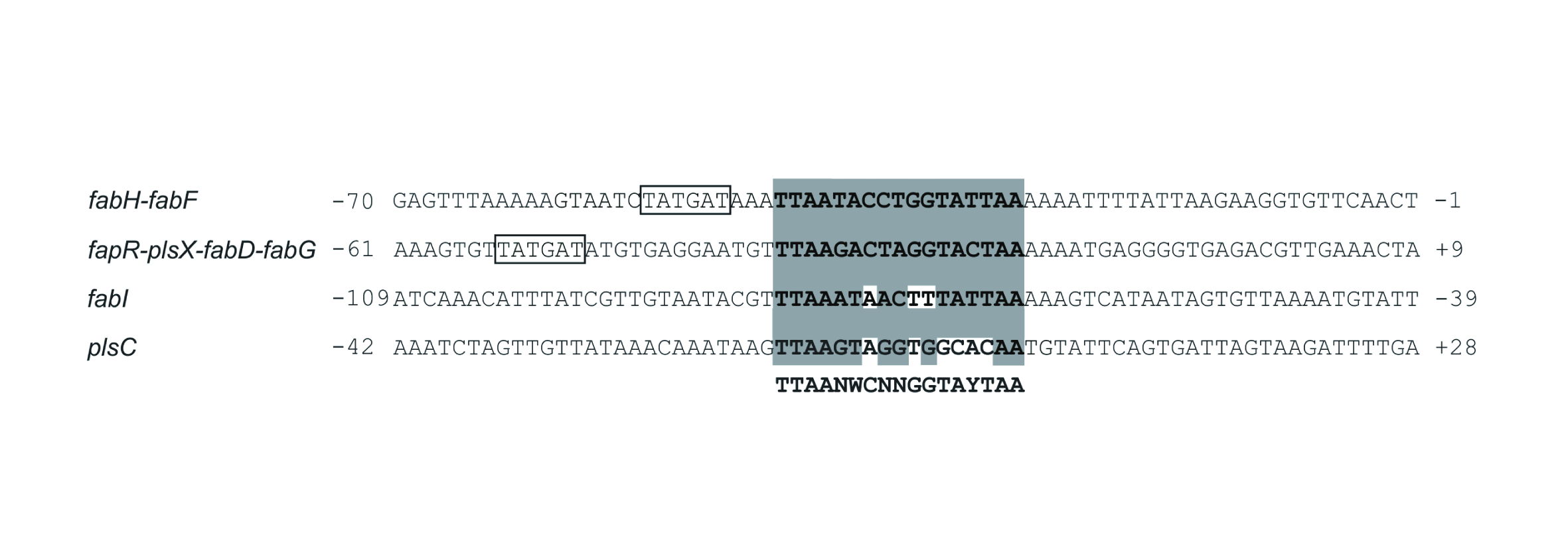

Supplement: Figure S4 — Alignment of the promoter regions of the genes and operons regulated by FapR. The -10 motifs of RNA polymerase binding boxes are indicated with rectangles. The conserved inverted repeat sequence is shaded and a consensus sequence is indicated at the bottom line. Bases are numbered related to the first base of the translation initiation codon. (TIF) [file ppat.1003108.s004.tif]

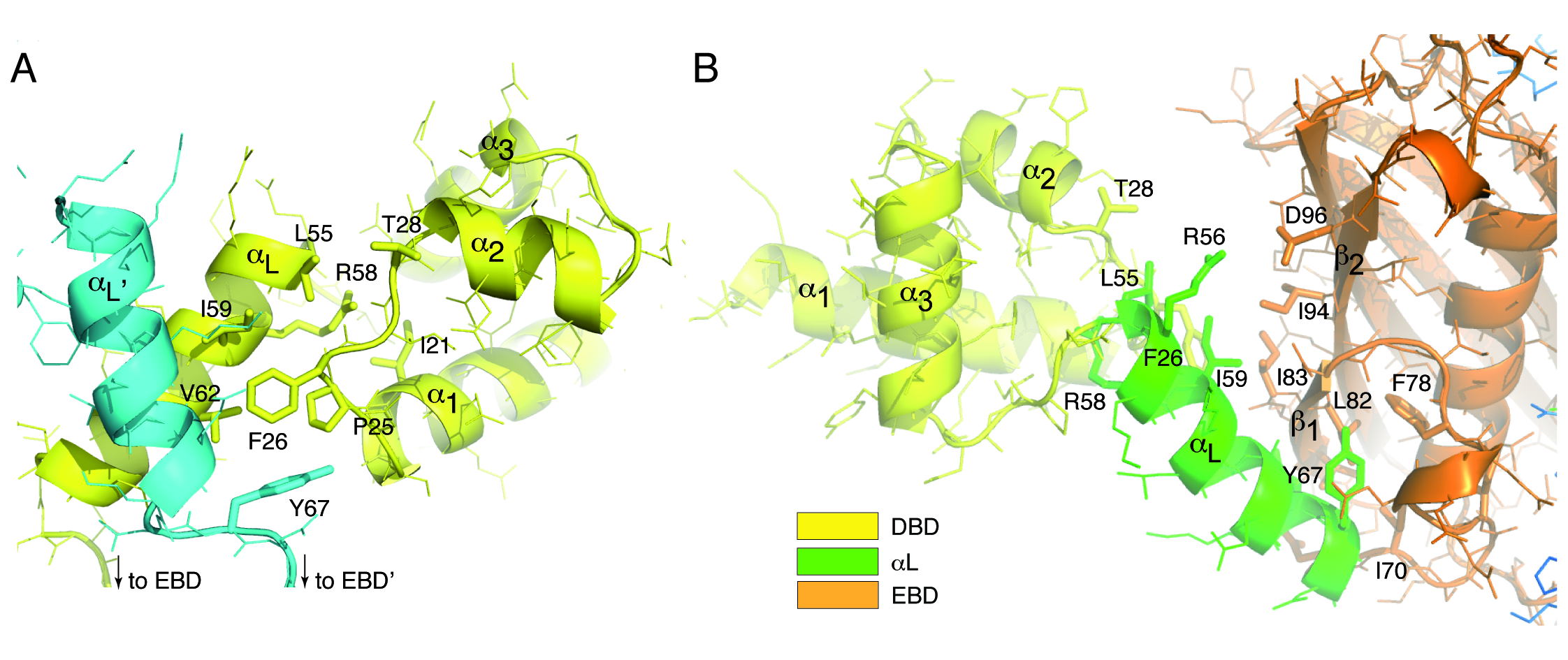

Supplement: Figure S5 — Different roles of the amphipatic linker helix αL in stabilizing the SaFapR-DNA and SaFapR-malonyl-CoA complexes. (A) Close view of the SaFapR-DNA complex. (B) Close view of the interactions between the EBD, the linker helix (green) and the DBD in the structure of the SaFapR-malonyl-CoA complex. In both cases, hydrophobic and hydrophilic side chains involved in inter-domain interactions are labeled. Malonyl-CoA binding triggers significant conformational changes in three main regions of the repressor: the connecting loop αL - β1, the amphipathic helix αL and the DBD. The side-chain of Arg110 from the EBD makes a key hydrogen bond with the main-chain carbonyl group of Glu77′ in the loop. In addition, the Glu77′ side-chain is hydrogen bonded to the main chain of His104 from the EBD, facilitating the formation of a strong salt bridge between the side-chains of Lys105 and Glu74′. The Ser102 side-chain makes a strong hydrogen bond with Val123, whereas its main chain interacts through hydrogen bonding networks with Ser72, Pro129 and a water molecule. Furthermore, the main-chain of Gln69 makes an electrostatic interaction with the side-chain of Lys127. These newly formed electrostatic/hydrogen-bond interactions allow the connecting loop αL - β1 to firmly interact with the core of the EBD. As a consequence, the amphipathic helix αL forms now an extended hydrophobic interface (Leu55, Ile59 and Val62), with both β1 (Ile83) and β2 (Ile94) strands of the EBD, and the α1–α2 connecting loop of the DBD (Pro25, Phe26 and Thr28). This structural arrangement is further stabilized by key electrostatic interactions. Arg56 makes a salt bridge with Asp96 located at the top of β2 and the side-chain of Asn66 makes a hydrogen bond with the carbonyl group of the main-chain of Leu82 on the β1 strand. Moreover, the side-chain of Tyr67 is oriented towards a hydrophobic pocket including residues Leu82, Val85 from β1, and Ile70, Ile73 and Phe78 from the connecting loop αL - β1. (TIF) [file ppat.1003108.s005.tif]

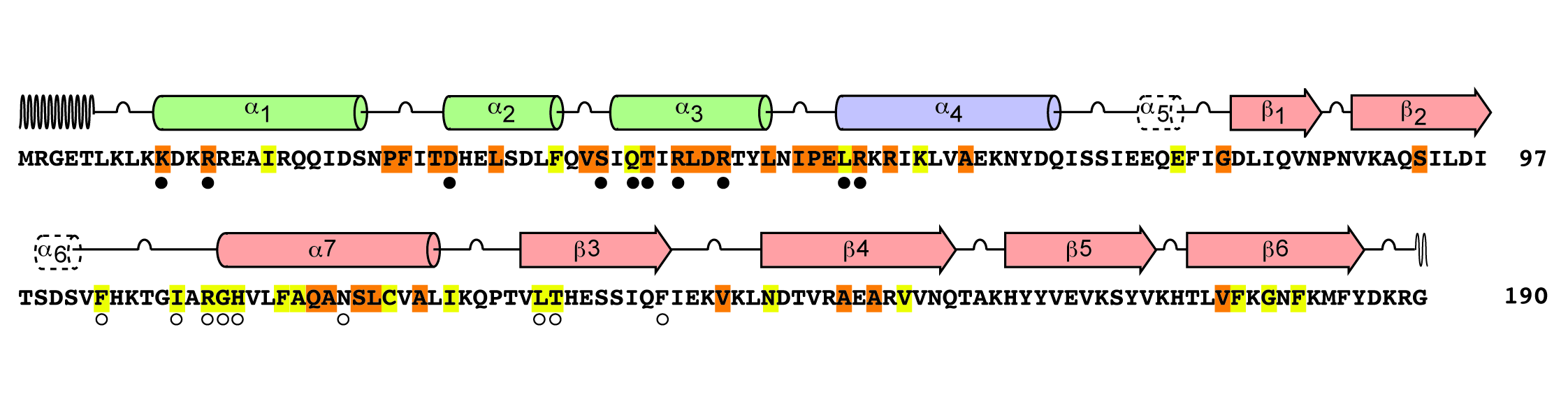

Supplement: Figure S6 — Sequence alignment of FapR. Amino acid sequence and secondary structure elements (green for DBD, pink for EBD) of SaFapR. Residues that interact with the pantetheine group of malonyl-CoA and with DNA are underlined by open and closed circles, respectively. Wavy lines indicate disordered regions in the crystal structure. Colored amino acid positions indicate highly conserved residues in non-redundant FapR sequences from 67 different bacterial species (yellow, >80%; orange, >95%). (TIF) [file ppat.1003108.s006.tif]

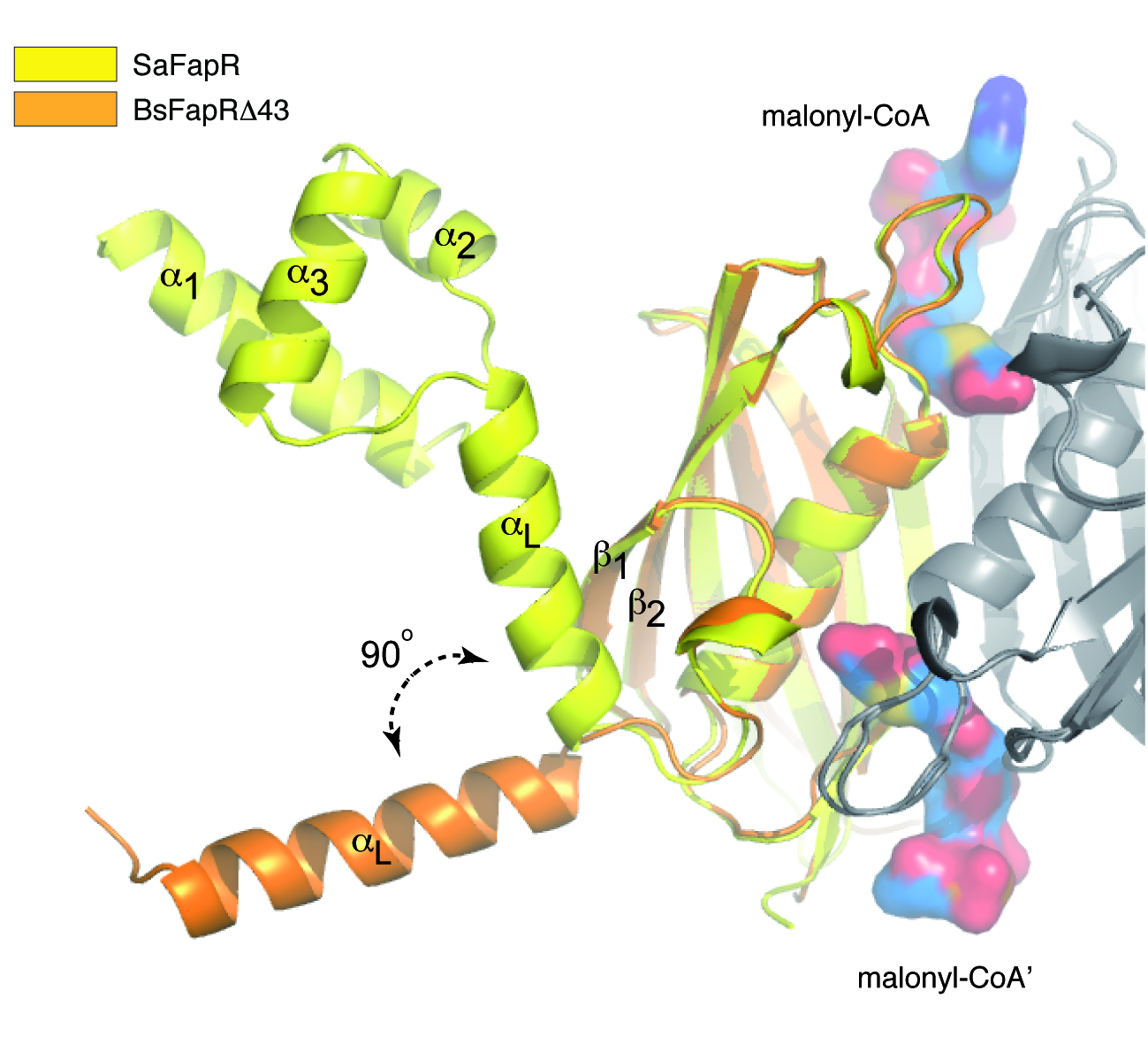

Supplement: Figure S7 — Structural superposition of the BsFapRΔ43-malonyl-CoA and SaFapR-malonyl-CoA complexes. Although the orientation of helix αL is different (it is involved in crystal packing contacts in BsFapRΔ43), binding of malonyl-CoA promotes the same conformation of the loop connecting this helix with the first β-strand of the EBD. (TIF) [file ppat.1003108.s007.tif]
