# Supplementary material for: Structural Basis for Feed-Forward Transcriptional Regulation of Membrane Lipid Homeostasis in Staphylococcus aureus
Source: PLoS Pathog. 2013 Jan 3;9(1):e1003108. doi: 10.1371/journal.ppat.1003108 (PMC3536700; doi:10.1371/journal.ppat.1003108)
Supplement: Table S1 — The fap regulon in B. subtilis and S. aureus. (DOC) [file ppat.1003108.s008.doc]

**SUPPORTING INFORMATION**

**Table S1**. The *fap* regulon in *B.* *subtilis* and *S. aureus^a^.*

| **Gene name and operon organization** | | **Gene function** |
| --- | --- | --- |
| ***Bacillus subtilis*** | ***Staphylococcus aureus*** |
| *fabHA-fabF* | *fabH-fabF* | β-ketoacyl-ACP synthaseIII, β-ketoacyl-ACP synthaseII |
| *fabHB* | absent | β-ketoacyl-ACP synthaseIII |
| *fapR-plsX-fabD-fabG* | *fapR-plsX-fabD-fabG* | transcriptional represor, phosphate:acyl-ACP acyltransferase, malonyl-CoA transacylase, β-ketoacyl-ACP reductase |
| *fabI* | *fabI* | enoyl reductase |
| *plsC* | *plsC* (SAR1804 in MRSA252) | 1-acylglycerol-phosphate (1-acyl-G3P) acyltransferase |
| *yhfC* | absent | unknown |

^a^ FapR controls the expression of eight genes involved in fatty acid or phospholipid biosynthesis in *B. subtilis*, which together constitute the *fap* regulon. Many of these genes, including *fapR*, are conserved and organized in a similar way in *S. aureus.*
