# Supplementary material for: Structural Basis for Feed-Forward Transcriptional Regulation of Membrane Lipid Homeostasis in Staphylococcus aureus
Source: PLoS Pathog. 2013 Jan 3;9(1):e1003108. doi: 10.1371/journal.ppat.1003108 (PMC3536700; doi:10.1371/journal.ppat.1003108)
Supplement: Table S2 — Local base-pair step parameters of the DNA in complex with SaFapR. (DOC) [file ppat.1003108.s009.doc]

**SUPPORTING INFORMATION**

**Table S2**. Local base-pair step parameters of the DNA in complex with *Sa*FapR.

| **Step *** | **Shift (Å)** | **Slide (Å)** | **Rise (Å)** | **Tilt (°)** | **Roll (°)** | **Twist (°)** |
| --- | --- | --- | --- | --- | --- | --- |
| -9 A-T | 0.07 | -0.17 | 3.10 | 2.56 | 2.37 | 33.84 |
| -8 T-A | 0.87 | -0.85 | 3.18 | 0.52 | 2.20 | 31.05 |
| -7 T-A | 0.10 | -0.65 | 3.27 | 1.44 | -0.17 | 34.38 |
| -6 A-T | 0.13 | -1.22 | 3.18 | 2.18 | 11.88 | 28.86 |
| -5 G-C | 0.42 | -0.72 | 3.13 | -5.58 | 1.99 | 28.34 |
| -4 T-A | -1.27 | -0.63 | 3.30 | 1.58 | 1.41 | 34.83 |
| -3 A-T | -0.06 | -0.05 | 3.05 | -1.65 | -0.04 | 33.88 |
| -2 C-G | 0.78 | -1.14 | 3.42 | -0.65 | 6.43 | 35.99 |
| -1 C-G | -0.51 | -1.22 | 3.79 | 1.18 | 4.01 | 29.71 |
| 0 T-A | -0.34 | 0.14 | 3.06 | 2.99 | 8.35 | 24.89 |
| 1 A-T | 0.58 | 1.01 | 3.07 | -1.38 | 10.60 | 36.00 |
| 2 G-C | 0.77 | -1.27 | 3.76 | -4.09 | 4.41 | 25.25 |
| 3 T-A | -0.87 | -1.71 | 3.23 | 0.84 | 6.61 | 33.95 |
| 4 C-G | 0.71 | -0.54 | 3.10 | 0.21 | 4.94 | 36.03 |
| 5 T-A | 0.05 | -0.69 | 3.14 | 1.06 | 5.48 | 22.97 |
| 6 T-A | -0.21 | -0.24 | 3.15 | -0.60 | 2.57 | 36.68 |
| 7 A-T | 0.19 | -0.57 | 3.47 | -0.30 | -1.43 | 34.76 |
| 8 A-T | 0.28 | -0.69 | 2.99 | -0.15 | -6.36 | 33.09 |
| 9 T-A | -0.33 | -0.63 | 3.43 | 0.60 | -0.76 | 33.84 |

*Base numbering as in Figure 1.
