# Supplementary material for: Structural Basis for Feed-Forward Transcriptional Regulation of Membrane Lipid Homeostasis in Staphylococcus aureus
Source: PLoS Pathog. 2013 Jan 3;9(1):e1003108. doi: 10.1371/journal.ppat.1003108 (PMC3536700; doi:10.1371/journal.ppat.1003108)
Supplement: Table S3 — Oligonucleotides used in this work. (DOC) [file ppat.1003108.s010.doc]

**SUPPORTING INFORMATION**

**Table S3.** Oligonucleotides used in this work

| Name | Sequence |
| --- | --- |
| OMD324 | 5’-GAAGAATTCGCGGTCGTGTAGGTAGAAGTG-3’ |
| OMD325 | 5’-CTCCTCGAGTTTCAACGTCTCACCCCTC-3’ |
| OMD326 | 5’-CTCCTCGAGGATAAGCGAGGATAAAATTATG-3’ |
| OMD327 | 5’-GGAGGATCCGGACCATGTTCCCAGTATAGC-3’ |
| OMD340 | 5’-GGTGTTTCCCGTGTAGGGTTATTGCATGG-3’ |
| OMD341 | 5’-CTGAACCACCGTATTCTGAGTAATCCATC-3’ |
| OMD342 | 5’-GAAGCCGTACAAAAGGCTGTT-3’ |
| OMD343 | 5’-CTGCTTCACCAGATTTCACAG-3’ |
| OMD346 | 5'-GGGCAGATGACGATCAAGATAC-3' |
| OMD349 | 5'-GATCCATAGAGGCAACTTTGCC-3' |
| fapRSauHind | 5’-ATATGTGAGGAAGCTTTAAGACTAGGTACT-3’ |
| fapRSauBam | 5’-TTTTGTACGGATCCTAATACGATATCA-3’ |
| TevFapRSauB_UP | 5’-CTTGGATCCTGAAAACCTGTATTTTCAGGGCATGAGGGGTGAGACGTTG3’ |
| FapRSauBH_DW | 5’-AAAGGATCCAAGCTTATTGTCGCCACCCATATC-3’ |
| *PfapR3* | 5’-ACTTCGTTAATTGAGTGATATGACAC-3’ |
| *PfapR4* | 5’-TAGTTCATGGTCTGTGATGAAGGG-3’ |
