# Supplementary material for: Structural Basis for Feed-Forward Transcriptional Regulation of Membrane Lipid Homeostasis in Staphylococcus aureus
Source: PLoS Pathog. 2013 Jan 3;9(1):e1003108. doi: 10.1371/journal.ppat.1003108 (PMC3536700; doi:10.1371/journal.ppat.1003108)
Supplement: Text S1 — Supplemental information, including additional experimental procedures. (DOC) [file ppat.1003108.s011.doc]

**SUPPORTING INFORMATION**

**Structural basis for feed-forward transcriptional regulation of membrane lipid homeostasis in *Staphylococcus aureus***

**Supplemental Methods**

***Strains and plasmid construction.*** Plasmids were constructed using standard methods and amplified in *E. coli* DH5α and XL1-Blue strains unless stated. Oligonucleotides used in this work are listed in Table S3. To construct an in frame deletion of *fapR* in strains RN4220 and HG001 [1] of *S. aureus*, upstream and downstream parts of the gene were amplified by PCR using oligonucleotides OMD324/OMD325 and OMD326/OMD327, respectively. The two PCR fragments (585 bp and 725 bp) were treated with *Xho*I and ligated afterwards. The ligation mixture was re-amplified by PCR using the lateral oligonucleotides OMD324 and OMD327 and the bipartite DNA product (1300 bp) was digested with *Eco*RI and *Bam*HI and cloned into the pMAD vector [2]. The recombinant plasmid pMAD-Δ*fapR* was introduced by electroporation into RN4220 and HG001 and erythromycin (erm) resistant clones were isolated at 30°C on TSA erm plates. Allelic exchange was performed as described [2] and candidate clones were identified by PCR using oligonucleotides OMD340 and OMD341. Amplification from the wild type strain leads to a DNA fragment of 2160 bp while the expected Δ*fapR* clones give a PCR product of 1625 bp. RN4220_Δ_*_fapR_* and HG001_Δ_*_fapR_* clones were obtained, reisolated and characterized. To test if *Sa*FapR was able to complement a *B. subtilis* Δ*fapR* strain, a DNA fragment containing full length *fapR* and the corresponding ribosome binding sequence was PCR amplified from *S. aureus* RN4220 genomic DNA using oligonucleotides fapRSauHind and fapRSauBam (Table S3). The PCR product was digested with *Bam*HI and *Hind*III and cloned into pGES247 (derivative of pDG1730 [3] containing *xylR* and P*xylA*), downstream of the P*xylA* promoter, to give plasmid pGES408. This plasmid was transformed into GS366, a *B. subtilis* Δ*fapR* strain containing a P*fabHB*-*lacZ* fusion ectopically integrated at the *amyE* locus [4], to give strain GS416.

The ORFs corresponding to wild-type *S. aureus fapR* and the two point mutants impaired in malonyl-CoA binding, *fapR_R110A_* and *fapR_G111V,L132W_* (including their ribosome binding sites) were PCR amplified using oligonucleotides fapRSauHind and fapRSauBam and the products digested with *Hind*III and *Bam*HI. In parallel, we obtained an *EcoR*I-*Hind*III DNA fragment from a plasmid (pGES483) containing the LacI repressor and the tight IPTG regulated P*spacOid* promoter from pMUTIN4. A triple ligation was then performed, including the above fragments and pOS1 digested with *EcoR*I and *Bam*HI, resulting in plasmids pOS1-LacIP*spacOid*-*fapR_WT_*, pOS1-LacI*PspacOid*-*fapR_R110A_* and pOS1-LacIP*spacOid*-*fapR_G111V,L132W_*.

For recombinant protein expression, *fapR* was PCR-amplified using oligonucleotides TevFapRSauB_UP and FapRSauBH_DW (Table S3) and plasmid pGES408 as template, digested with *BamH*I and cloned into the expression vector pET15b (Novagen). The recombinant protein includes a Tobacco Etch Virus (TEV) protease cleavage site for N-terminal His-tag removal. All the constructs were confirmed by DNA sequence analysis.

***RNA preparation***. *S. aureus* strains RN4220, RN4220_Δ_*_fapR_*, HG001 and HG001_Δ_*_fapR_* were grown in TSB at 37°C with aeration to an OD_600_ of 1.0. The cells were recovered by centrifugation and immediately frozen at –20°C. RNA extractions were performed as previously described [5] and treated with the TURBO DNA-free reagent (Ambion, Austin, TX).

***Real Time PCR***. For real time quantitative experiments we used oligonucleotides OMD342 and OMD343 for *pslX*, and OMD346 and OMD349 for *fabH* (Table S3). Real-time PCRs (qRT-PCRs) were performed in a 25-µl reaction volume containing 5 µl of a 1/100 dilution of cDNA, 1 µl of gene-specific primers (10 µM), and 12.5 µl of iQ SYBR Green Supermix (Bio-Rad, Hercules, CA). PCR amplification, detection, and analysis were performed with the MyiQ Single-Color Real-Time iCycler PCR Detection System and the MyiQ Optical System Software (Bio-Rad, Hercules, CA). PCR conditions included an initial denaturation step at 95°C for 3 min, followed by a 40-cycle amplification (95°C for 15 s, 55°C for 15 s, and 72°C for 15 s). The specificity of the amplified product and the absence of primer dimer formation were verified by generating a melting curve with a final step of 80 cycles consisting of a stepwise 0.5°C temperature increase every 10 s, beginning at 55°C. The absence of contaminating genomic DNA was verified by testing each sample in control reactions without a prior reverse transcription step. The critical threshold cycle (CT) was defined for each sample. The expression levels of the tested genes were normalized using the 16S rRNA gene of *S. aureus* as an internal standard whose transcript level did not vary under our experimental conditions. Each assay was performed in quadruplicate and repeated with at least three independent RNA samples, obtained as indicated in the SI section. The change (n-fold) in the transcript level was calculated using the following equations: ΔCT = CT(test DNA) – CT(reference cDNA), ΔΔCT = ΔCT(target gene) – ΔCT(16S rRNA), and ratio = 2–ΔΔCT [6].

***Gel shift assays.*** A 300 bp DNA fragment containing the P*fapR* promoter region was prepared by PCR using primers *PfapR3* and *PfapR4* and *S. aureus* RN4220 genomic DNA as template. The PCR product was subsequently purified from a 2 % agarose gel. Binding of purified *Sa*FapR to DNA fragments was carried out in a 10 µl reaction mix containing 20 mM Tris-HCl, 350 mM NaCl, 10 mM MgCl_2_, 5 mM DTT, pH 8. After incubation for 15 min at room temperature, 0.6 µl of 50% glycerol were added and the samples were loaded onto a native 5% polyacrylamide gel which had been pre-run for 2 hs in 45 mM Tris-borate pH 8, 1 mM EDTA. To test the effect of different acyl-CoAs on complex formation, *Sa*FapR was preincubated for 15 min at room temperature in the presence of the molecule of interest and then the double stranded DNA was added. In all cases gels were stained with SYBR GREEN (Invitrogen) and scanned at 530 nm using a phosphoimager Storm Byo (Amersham Biosciences).

***Crystallization.*** Initial crystallization screenings were carried out at 18 °C by the vapor diffusion method using a Cartesian nanolitre dispensing system; hits were improved by hand-made hanging drops in 24-well plates. Crystals of the *Sa*FapR-DNA complex were obtained with a high quality double-stranded oligonucleotide (EUROGENTEC) containing the FapR operator site: 5’-GCCAATTATATACTACTATTAGTACCTAGTCTTAATTCCG-3’. To promote complex formation, *Sa*FapR (15 mg/ml) was slowly added to the DNA stock solution considering a 4:1 molar ratio (protein:DNA) for binding, as indicated by ITC experiments, and including an excess of 10% DNA. The mixture was incubated at room temperature for 1 hour prior to crystallization. Crystals of apo-*Sa*FapR (10 mg/ml) were optimally grown in 6% PEG-8000, 0.1M MES, 0.2 M Zn Acetate, pH=5.4 (crystal form 1) or 3.5 M NaCl, 0.1 mM Hepes pH=7.5 (crystal form 2); those of the *Sa*FapR-malonyl-CoA complex (15 mg/ml *Sa*FapR incubated with 2.5 mM malonyl-CoA) in 30% MPD, 5% PEG-4000, 0.1M Na Hepes, pH=7.5; and the *Sa*FapR-DNA complex in 3.2 M Na formate.

**Supplemental Methods References**

1 Herbert S, et al. (2010) Repair of global regulators in *Staphylococcus aureus* 8325 and comparative analysis with other clinical isolates. *Infect Immun* 78:2877-2889.

2 Arnaud M, Chastanet A, Debarbouille M (2004) New vector for efficient allelic replacement in naturally nontransformable, low-GC-content, gram-positive bacteria. *Appl Environ Microbiol* 70:6887-6891.

3 Guerout-Fleury AM, Frandsen N, Stragier P (1996) Plasmids for ectopic integration in *Bacillus subtilis*. *Gene* 180:57-61.

4 Schujman GE, et al. (2006) Structural basis of lipid biosynthesis regulation in Gram-positive bacteria. *EMBO J* 25:4074-4083.

5 Chastanet A, Prudhomme M, Claverys JP, Msadek T (2001) Regulation of *Streptococcus pneumoniae* *clp* genes and their role in competence development and stress survival. *J Bacteriol* 183:7295-7307.

6 Livak KJ, Schmittgen TD (2001) Analysis of relative gene expression data using real-time quantitative PCR and the 2(-Delta Delta C(T)) Method. *Methods* 25:402-408.
